# Genetic Dissection of Azuki Bean Weevil (*Callosobruchus chinensis* L.) Resistance in Moth Bean (*Vigna aconitifolia* [Jaqc.] Maréchal)

**DOI:** 10.3390/genes9110555

**Published:** 2018-11-15

**Authors:** Prakit Somta, Achara Jomsangawong, Chutintorn Yundaeng, Xingxing Yuan, Jingbin Chen, Norihiko Tomooka, Xin Chen

**Affiliations:** 1 Institute of Industrial Crops, Jiangsu Academy of Agricultural Sciences, 50 Zhongling Street, Nanjing 210014, China; yundaeng@gmail.com (C.Y.); yxx@jaas.ac.cn (X.Y.); chenjingbin@jaas.ac.cn (J.C.); 2 Department of Agronomy, Faculty of Agriculture at Kamphaeng Saen, Kasetsart University, Kamphaeng Saen Campus, Nakhon Pathom 73140, Thailand; 3 Center for Agricultural Biotechnology (AG-BIO/PEDRO-CHE), Kasetsart University, Kamphaeng Saen Campus, Nakhon Pathom 73140, Thailand; 4 Program in Plant Breeding, Faculty of Agriculture at Kamphaeng Saen, Kasetsart University, Kamphaeng Saen Campus, Nakhon Pathom 73140, Thailand; pk_achara@hotmail.com; 5 Genetic Resources Center, Gene Bank, National Agriculture and Food Research Organization, 2-1-2 Kannondai, Tsukuba, Ibaraki 305-8602, Japan; tomooka@affrc.go.jp

**Keywords:** bruchid resistance, seed weevil, *Callosobruchus*, insect resistance, moth bean, QTL

## Abstract

The azuki bean weevil (*Callosobruchus chinensis* L.) is an insect pest responsible for serious postharvest seed loss in leguminous crops. In this study, we performed quantitative trait locus (QTL) mapping of seed resistance to *C. chinensis* in moth bean (*Vigna aconitifolia* [Jaqc.] Maréchal). An F_2_ population of 188 plants developed by crossing resistant accession ‘TN67’ (wild type from India; male parent) and susceptible accession ‘IPCMO056’ (cultivated type from India; female parent) was used for mapping. Seeds of the F_2_ population from 2014 and F_2:3_ populations from 2016 and 2017 were bioassayed with *C. chinensis*, and the percentage of damaged seeds and progress of infestation severity were measured. Segregation analysis suggested that *C. chinensis* resistance in TN176 is controlled by a single dominant gene, designated as *Rcc*. QTL analysis revealed one principal and one modifying QTL for the resistance, named *qVacBrc2.1* and *qVacBrc5.1*, respectively. *qVacBrc2.1* was located on linkage group 2 between simple sequence repeat markers CEDG261 and DMB-SSR160 and accounted for 50.41% to 64.23% of resistance-related traits, depending on the trait and population. Comparative genomic analysis suggested that *qVacBrc2.1* is the same as QTL *Brc2.1* conferring *C. chinensis* resistance in wild azuki bean (*V. nepalensis* Tateishi and Maxted). Markers CEDG261 and DMB-SSR160 should be useful for marker-assisted selection for *C. chinensis* resistance in moth bean.

## 1. Introduction

The genus *Vigna* is an important plant taxon that contains more than 10 species cultivated as human food and animal feed. Among *Vigna* species, cowpea (*Vigna unguiculata* [L.] Walp.), mungbean (*Vigna radiata* [L.] Wilczek), and black gram (*Vigna mungo* [L.] Hepper) are internationally well-known, economically important crops, especially in Africa and Asia. Other *Vigna* species, such as azuki bean (*Vigna angularis* [Willd.] Ohwi and Ohashi), rice bean (*Vigna umbellata* [Thunb.] Ohwi and Ohashi), and moth bean (*Vigna aconitifolia* [Jaqc.] Maréchal), are grown as minor crops in several Asian and African countries.

Bruchids or seed weevils (Coleoptera: Bruchidae) are a group of storage insects that feed on dry seeds of leguminous plants [1]. Infestation by bruchids initially occurs in the field, where female adult bruchids lay eggs on young pods; the larvae then bore through pods to the seeds, where they grow and develop into adults while consuming seed nutrients. After harvest, adult bruchids emerge from the seeds and start a secondary infestation by laying eggs directly on seeds. The first infestation usually leads to minor seed loss, while the secondary infestation can cause the total loss of a seed lot within 3 to 4 months [2]. The azuki bean weevil (*Callosobruchus chinensis* L.) and cowpea weevil (*Callosobruchus maculatus* L.) are serious bruchid pests of most *Vigna* species, especially cultivated ones. The azuki bean weevil is widely distributed in Asia, whereas the cowpea weevil is mainly found in Africa; however, both species are now additionally found in several other locations as a result of the international seed trade [1]. Although chemical fumigation can be used to control bruchids, this method is not practical for small-scale farmers and traders and is harmful to human health and the environment. Chemical control also increases production costs. The best way to manage bruchid infestation is by using resistant cultivars [3].

Plant breeders have long been interested in breeding cowpea, mungbean, azuki bean, and black gram for bruchid resistance. Although sources of bruchid resistance have been identified for these *Vigna* crops, resistant germplasm is rare. Three cowpea sources resistant to *C. maculatus* were reported by Singh [4], all of which were cultivated form and moderately resistant. Some cultivated mungbean (*V. radiata* var. *radiata*) and a few wild mungbean (*V. radiata* var. *sublobata*) germplasm (<10 accessions in total) have been found to be resistant to *C. chinensis* and *C. maculatus* [5,6,7,8]. No cultivated azuki bean (*V. angularis* var. *angularis*) or wild azuki bean (*V. angularis* var. *nipponensis*) germplasm with resistance to *C*. *chinensis* or *C*. *maculatus* have been identified [9]; however, an accession of *Vigna nepalensis* Tateishi and Maxted, which is very closely related to and cross-compatible with azuki bean, is able to reduce the severity of seed infestation by *C*. *chinensis* and *C*. *maculatus* [10]. Cultivated black gram (*V. mungo* var. *mungo*) is susceptible to *C*. *maculatus*, but its wild progenitor, *V. mungo* var. *silvestris*, is resistant to this bruchid [3].

Moth bean (also known as mat bean and dew bean) is one of the most heat- and drought-tolerant leguminous crops. Moth bean is grown in drought-prone, semi-arid, and arid areas of India, Afghanistan, Pakistan, Nepal, Sri Lanka, Myanmar, and some African countries. This crop can grow in harsh climates with daytime temperatures up to 45 °C and annual rainfall of 200 to 300 mm [11]. India is the largest producer of moth bean, with a production area of 1.5 Mha. The main cultivation area encompasses arid regions of the state of Rajasthan [11], where moth bean is the most widely grown drought-tolerant legume. Seeds and young pods of moth bean are consumed by humans, while leaves and stalks are used as animal feed in the form of forage and hay [12]. Dry seeds of moth bean contain 23% to 25% protein. Sprouts of moth bean seeds are also consumed as a vegetable for their vitamins and minerals.

Although moth bean is a highly heat- and drought-tolerant crop that greatly benefits impoverished people in arid regions, it is extremely susceptible to insect pests, including bruchids [13,14]. The bruchid species most damaging to moth bean are *C. chinensis* and *C. maculatus*. Breeding for resistance to these bruchids is an important goal in moth bean breeding, but no source of resistance had previously been identified for this crop. Recently, however, an accession of wild moth bean was found to be highly resistant to *C. chinensis*. The resistance gene(s) in wild moth bean is useful for breeding a new moth bean with *C. chinensis* resistance. In this study, we conducted the first-ever molecular genetic analysis of moth bean seed resistance to *C. chinensis*. Our objectives were two-fold: (i) to determine the mode of inheritance of moth bean seed resistance to C. *chinensis* and (ii) to locate quantitative trait loci (QTLs) for this resistance trait.

## 2. Materials and Methods

### 2.1. Plant Materials

An F_2_ population of 188 plants derived from self-pollination of a single F_1_ plant of a cross between ‘TN67’ and ‘IPCMO056’ was used in this study. In this cross, TN67 and IPCMO056 were used as male and female parents, respectively. The F_2_ population was previously used to construct a SSR-based linkage map and identify QTLs for domestication syndrome in moth bean [15]. TN67, a wild moth bean collected in India, is resistant to *C. chinensis*, while IPCMO056 is a cultivated moth bean from India that is susceptible to *C. chinensis*. Compared with susceptible moth bean accessions, TN67 exhibits fewer damaged seeds and a slower progress of seed damage due to *C. chinensis*.

F_2_ plants and four plants of each parent were grown under field conditions at the National Agricultural and Food Research Organization, Tsukuba, Japan, from July to September 2014. Spacing between plants was 1 m. Seeds of each F_2:3_ plant were harvested separately and used for bruchid resistance evaluation. In addition to the F_2_ population, the F_2:3_ population and parents were grown in a nonreplicated experiment during February to May (summer) of 2016 at the Chai Nat Field Crops Research Center (CNFRC), Chai Nat, Thailand. Each entry comprising 10 plants was grown in a 6-m-long row. Spacing between rows was 0.75 m, with plants in a row spaced 0.5 m apart. This population is hereafter referred to as F_2:3_-A. Seeds of each F_2:3_ plant were harvested separately. In addition, another F_2:3_ population (population F_2:3_-B) was grown together with the parents in a nonreplicated experiment during December 2016 to March 2017 at the CNFRC. Planting, spacing, and harvesting were the same as for population F_2:3_-A.

### 2.2. Evaluation of Seeds for Bruchid Resistance

*Callosobruchus chinensis* was used for seed resistance evaluation. The insects were reared on seeds of susceptible mungbean cultivar ‘Kamphaeng Saen 1’ and kept at 30 °C and 70% relative humidity (RH). Before evaluation for *C. chinensis* resistance, 100-seed weights of each plant were determined. Evaluation for *C. chinensis* resistance followed the method described by Somta [16] with minor modifications. In the F_2_ population, 30 to 40 seeds from each plant were placed in a plastic box. Ten pairs (10 males and 10 females) of newly emerged *C. chinensis* adults were then introduced into the box for egg laying and removed after 7 days. The infested seeds were maintained at 30°C and 60% RH. Seeds of each parent were included in the resistance evaluation, and two technical replicates was conducted. The number of bruchid-damaged seeds was counted at 23 days after infestation (DAI) and then every 3 days until 56 DAI. After each count, damaged seeds were removed from the boxes. The cumulative number of seeds damaged by bruchids at each time point was calculated and converted into a percentage. The percentage of damaged seeds was then used to calculate the area under the disease progress curve (AUDPC), which reflects the progress of the development of disease severity in plants [17]. AUDPC, which was thus an indicator of the progress of infestation severity (bruchid developmental period) in this study, was calculated as follows

$$AUDCP=\overset{n=1}{\underset{i=1}{\sum }}\frac{y_{i}+y_{i+1}}{2}\times \left(t_{i+1}-t_{i}\right)$$

where *y_i_* is the percentage of damaged seeds at the *i*^th^ observation, *t_i_* is the number of days at the *i*^th^ observation, and *n* is the total number of observations.

For the F_2:3_-A population, F_4_ seeds from each F_2:3_ plant and the parents were evaluated separately for resistance. Forty seeds of each plant were evaluated for resistance in the same manner as described for F_2_ plants, expect that the percentage of damaged seeds was only recorded at 60 DAI. Two technical replicates were performed. The average percentages of damaged seeds of each family were used for data analysis.

In the case of the F_2:3_-B population, two sets of seeds (I and II) were evaluated for resistance. In set I, only the percentage of damaged seeds was determined, whereas both this percentage and AUDPC were calculated in set II. In set I, 40 seeds of each plant per line were separately evaluated for resistance as described for F_2_ plants, except that the percentage of damaged seeds was determined at 60 DAI. The average percentage of damaged seeds of each line was used for data analysis. In set II, seeds of all plants of a given line (10 seeds each) were bulked and evaluated for bruchid resistance as described for F_2_ plants with the following modifications, the percentage of damaged seeds was determined at 60 DAI, and the number of bruchid-damaged seeds was counted at 25 DAI and then every 5 days until 60 DAI.

### 2.3. Correlation Analysis and Determination of the Mode of Inheritance of Resistance

Correlations between the percentage of damaged seeds and AUDPC and between the percentage of damaged seeds/AUDPC and 100-seed weight were assessed using R 2.0.10 [18].

The mode of inheritance of bruchid resistance was determined in the F_2_ and F_2:3_ populations for two traits: percentage of damaged seeds and AUDPC. On the basis of the percentage of damaged seeds, F_2_ plants and F_2:3_ lines were classified as resistant or susceptible following Somta et al. [16]. F_2_ plants and F_2:3_ lines having 0% to 80% damaged seeds were considered to be resistant, which included both homozygous resistant (highly resistant, with 0% to 20% damaged seeds) and heterozygous resistant (moderately resistant, with 21% to 80% damaged seeds) genotypes, while those having 81% to 100% damaged seeds were considered as susceptible. Chi-square (χ^2^) testing was conducted to determine the 3:1 (resistant:susceptible) ratio goodness of fit under a single gene model using R 2.0.10 [18].

To examine the inheritance of AUDPC, the F_2_ plants and F_2:3_ lines were also classified into two categories. Plants/lines showing AUDPs of 0 to 2040 were classified as resistant, while those showing AUDPC values higher than 2040 were considered to be susceptible. A χ^2^ test was conducted as described above.

### 2.4. Estimation of the Heritability of Resistance

The broad-sense heritability (*H*^2^) of the percentage of damaged seeds and/or AUDPC in F_2_, F_2:3_-A, and F_2:3_-A populations was calculated according to the formula

$$H^{2}=\frac{\sigma_{F_{2}}^{2}-\left(\frac{\sigma_{P_{1}}^{2}+\sigma_{P_{2}}^{2}}{2}\right)}{\sigma_{F_{2}}^{2}}h^{2}=\frac{\sigma_{F_{2}}^{2}-\left(\frac{\sigma_{P_{1}}^{2}+\sigma_{P_{2}}^{2}}{2}\right)}{\sigma_{F_{2}}^{2}}$$

where $\sigma_{F_{2}}^{2}$ is the variance of the F_2_ or F_2:3_ population, $\sigma_{P_{1}}^{2}$ is the variance of ICPMO056 and $\sigma_{P_{2}}^{2}$ is the variance of TN67.

### 2.5. Quantitative Trait Loci Analysis

The F_2_ population used in this study was previously genotyped with 169 simple sequence repeat (SSR) and three morphological markers, which were used to construct a linkage map containing 11 linkage groups (LGs) (Yundaeng et al., 2018) [15]. The map and genotypic data were used for QTL analysis in the present study. The percentage of damaged seeds and AUDPC were applied to locate QTLs for bruchid resistance. The QTL analysis was conducted using the inclusive composite interval mapping (ICIM) method [19] as implemented in the program QTL IciMapping 4.1. A walking speed of 1 cM and a probability in stepwise regression (PIN) of 0.001 were used for the ICIM. Significant logarithm of odds (LOD) thresholds for QTLs for each trait were determined by a 10,000 permutation test at *p* = 0.001.

In addition to QTLs for bruchid resistance, QTLs for seed weight were also identified in the F_2_ and F_2:3_ populations by ICIM using the same procedures as for bruchid resistance. This analysis was conducted to determine the genetic relationship between seed weight and bruchid resistance.

### 2.6. Comparative Genomic Analysis of Bruchid-Resistance QTLs in Moth Bean, Mungbean and Wild Azuki Bean Relatives

The genomic region harboring detected QTLs for *C. chinensis* resistance in moth bean were compared with reported genes/QTLs for *C. chinensis* resistance in mungbean (*V. radiata*) [20] and *V. nepalensis* [10], a wild form of azuki bean (*V. angularis*), using common DNA markers and DNA marker locations on physical maps of mungbean and azuki bean. The physical locations of DNA markers were determined by BLASTN searching against reference genome sequences of mungbean ([21] http://plantgenomics.snu.ac.kr/mediawiki-1.21.3/index.php/Main_Page) and azuki bean ([22]; http://viggs.dna.affrc.go.jp). The *C. chinensis* resistance QTLs in moth bean were also compared with reported genes/QTLs for *C. maculatus* resistance in black gram (Souframanien et al., 2010) and rice bean [23].

## 3. Results

### 3.1. Variation in *Callosobruchus chinensis* Resistance in Parents and F_2_ and F_2:3_ Generations

**Figure 1:**
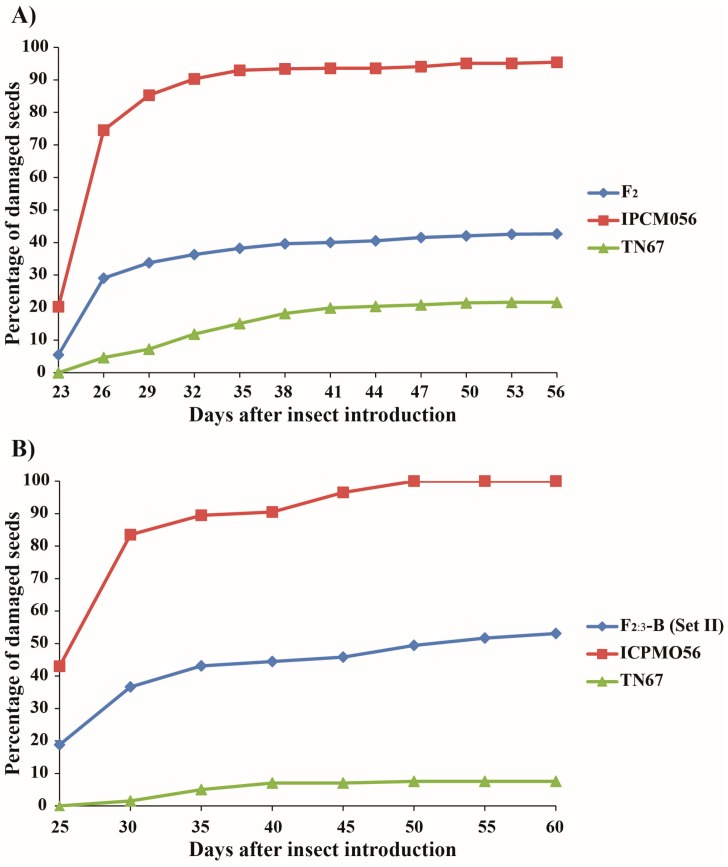
Patterns of *Callosobruchus chinensis* infestation of seeds of moth bean accessions TN67 and IPCMO056 and their derivative F_2_ population as reflected by the percentage of damaged seeds (**A**) and the area under the disease progress curve (AUDPC) (**B**).

TN67 and IPCMO056 exhibited contrasting phenotypes for two traits related to *C. chinensis* resistance, namely, the percentage of damaged seeds and the progress of damage severity (the developmental period of bruchid adults) (Figure 1). With respect to the first trait, TN67 had a much lower percentage of damaged seeds than IPCMO056, with values of 6.6% to 21.6% vs. 95.4% to 100%, respectively, depending on the growing environment (Table 1). The average value of this trait across growing environments and tests was 11.9% for TN67 and 98.1% for IPCMO056. The percentage of damaged seeds in the F_2_ population ranged from 0% to 100%, with an average of 41.6% (Table 1). Similarly, the percentage of damaged seeds in the F_2:3_-A population ranged from 3.5% to 99.6%, with an average of 57.0% (Table 1). Two sets of seeds were evaluated for resistance in the F_2:3_-B population; the percentage of damaged seeds in set I varied between 0.5% to 100%, with an average of 53.1%, while that of set II ranged between 1.0% to 100%, with an average of 52.2% (Table 1).

In terms of the progress of damage severity, AUDPCs of TN67 and IPCMO056 were correspondingly 197.0 to 500.0, with an average of 348.5, and 2826.0 to 3158.0, with an average of 2992.0 (Table 1). This result indicates that seeds of TN67 were damaged much more slowly than those of IPCMO056 (a longer bruchid adult developmental period). AUDPC in the F_2_ population ranged from 0 to 3103.1, with average of 1163.2, while that in the F_2:3_-B population varied from 16.4 to 3385.0, with an average of 1536.7 (Table 1).

**Figure 2:**
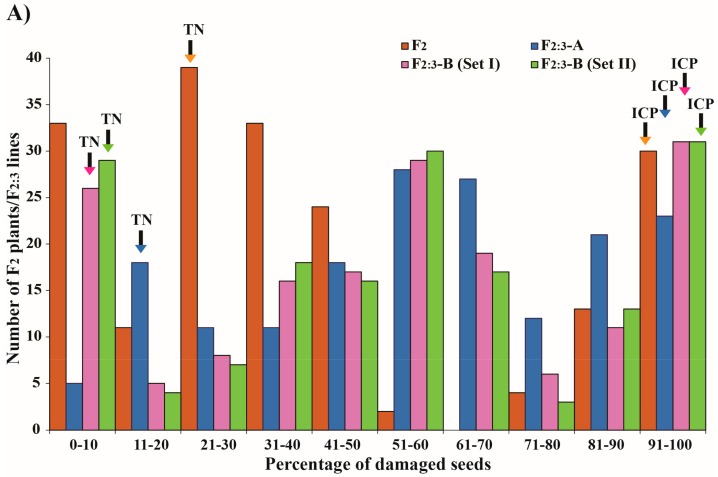

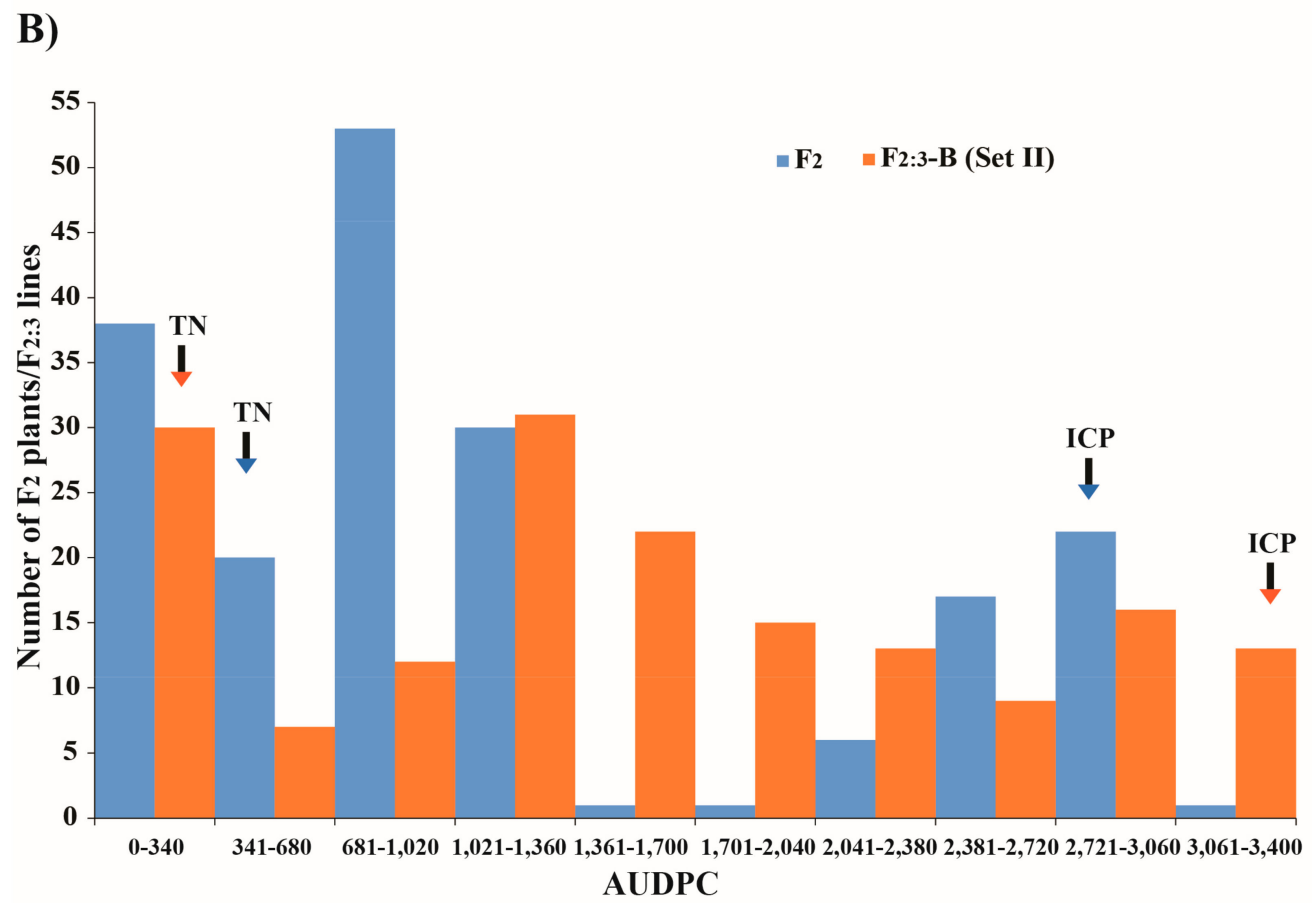
Frequency distribution of the percentage of damaged seeds (**A**) and the AUDPC (**B**) due to *C. chinensis* in moth bean F_2_ and F_2:3_ populations derived from an IPCMO056 × TN67 cross.

The frequency distribution of the percentage of damaged seeds was trimodal and discontinuous in the F_2_ population, but was trimodal and continuous in the F_2:3_ ones (Figure 2A). Similarly, AUDPCs in the F_2_ and F_2:3_ populations displayed trimodal and continuous distribution patterns (Figure 2B). The frequency distribution of both traits suggests that resistance to *C. chinensis* in moth bean TN67 is controlled by one or a few genes with quantitative expression.

### 3.2. Correlations among Traits

A nearly perfect correlation was observed between the percentage of damaged seeds and AUDPC in both the F_2_ and F_2:3_-B populations: *r* = 0.99 (d.f. = 185, *p* < 0.0001) and *r* = 0.99 (d.f. = 164, *p* < 0.0001), respectively. This result suggests that these two traits are controlled by the same gene(s).

A low but significant positive correlation was found between the percentage of damaged seeds and 100-seed weight in the F_2_ population (*r* = 0.22, d.f. = 185, *p* = 0.0028), but no significant correlation between the two traits was detected in the F_2:3_-A and F_2:3_-B populations (*r* = 0.07, d.f. = 151, *p* = 0.4070 and *r* = 0.11, d.f. = 164, *p* = 0.1693, respectively). Similarly, a weak but significant positive correlation was observed between AUDPC and 100-seed weight in the F_2_ population (*r* = 0.23, d.f. = 185, *p* = 0.0014), but no significant correlation was found between these traits in the F_2:3_-B population (*r* = 0.10, d.f. = 164, *p* = 0.1789, respectively).

### 3.3. Segregation Analysis and Heritability of Resistance

A χ^2^ test for the percentage of damaged seeds revealed that the segregation of this trait in the F_2_ population and in an F_2:3_ population grown in 2016 and 2017 fit a 3:1 (resistance: susceptible) ratio (Table 2). In the case of AUDPC, a χ^2^ test indicated that this trait segregated in a 3:1 (resistance: susceptible) ratio in both the F_2_ and F_2:3_ populations (Table 2). These results suggest that seed resistance to *C. chinensis* in moth bean TN67 is controlled by a single dominant locus. We accordingly named this locus *Resistance to Callosobruchus chinensis* (*Rcc*).

The calculated broad-sense heritability of the percentage of damaged seeds in F_2_, F_2:3_-A, and F_2:3_-B (sets I and II) populations was very high, varying from 83.60% to 99.97% (Table 1). The heritability calculated for AUDPC in F_2_ and F_2:3_-B (set I) populations was also very high, 85.99% and 98.65%, respectively (Table 1). These results indicate that seed resistance to *C. chinensis* in moth bean TN67 is principally controlled by one or more genetic factors.

### 3.4. QTL Analysis

**Figure 3:**
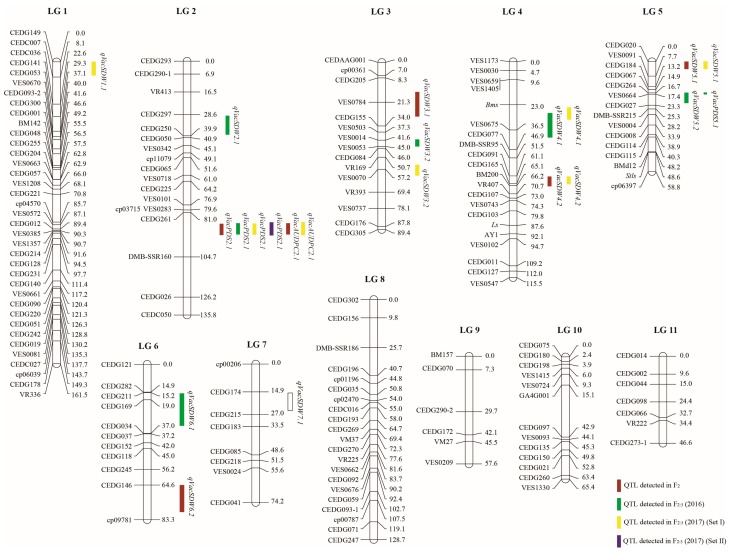
Linkage map showing the location of Quantitative trait loci (QTLs) for percentage of damaged seeds and the AUDPC caused by *Callosobruchus chinensis* and for 100-seed weight in moth bean F_2_ and F_2:3_ populations derived from the cross IPCMO056 × TN67. The QTLs were detected by inclusive composite interval mapping (ICIM).

**Figure 4:**
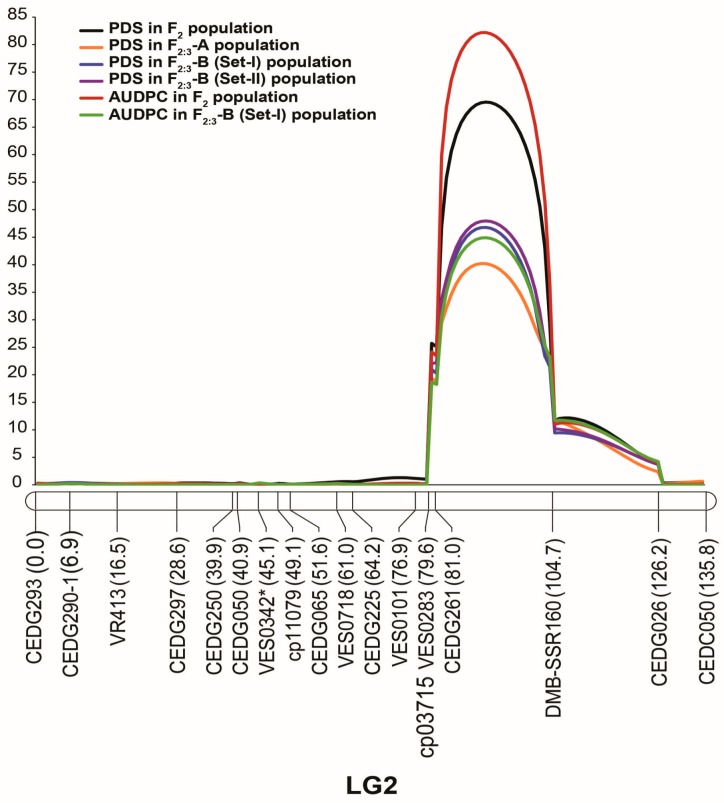
Logarithm of odds (LOD) graph of QTLs for percentage of damaged seeds (PDS) and the AUDPC on linkage group 2 detected by ICIM in moth bean F_2_ and F_2:3_ populations derived from the cross TN67 × IPCMO056.

ICIM identified two QTLs for the percentage of damaged seeds (Table 3, Figure 3 and Figure 4). These QTLs were named *qVacPDS2.1* and *qVacPDS5.1*. *qVacPDS2.1* was consistently identified in all populations/environments, while *qVacPDS5.1* was identified in only one population. *qVacPDS2.1* was located between 90 and 91 cM and was flanked by markers CEDG261 and DMB-SSR160 on LG2. This QTL accounted for 50.41% to 64.23% of the variation in the percentage of damaged seeds; it showed an additive effect of 32.91% to 42.64% and a dominant effect of 0.10% to −11.16%. *qVacPDS5.1* was located at 17 cM between markers CEDG264 and VES0664 on LG5; it explained 12.19% of the trait variation and showed an additive effect of 0.51% and a dominant effect of 22.76%. At both QTLs, one or more alleles from IPCMO056 decreased the percentage of damaged seeds. 

In the case of AUDPC, which was evaluated in F_2_ and F_2:3_-B (set I) populations, ICIM consistently identified a single major QTL for this trait (Table 3, Figure 3 and Figure 4). We named this QTL *qVacAUDPC2.1*. The position of *qVacAUDPC2.1* was the same as that of *qVacPDS2.1*. *qVacAUDPC2.1* explained 50.41% and 58.78% of the total AUDPC variation in the F_2_ and F_2:3_-B (set I) populations, respectively; it possessed additive effects of 1273.19 and 1279.74 and dominant effects of −483.39 and −90.70 in the F_2_ and F2:3-B (set I) populations, respectively. At this QTL, one or more alleles from IPCMO056 decreased the AUDPC value. Given that the locations and effects of QTLs *qVacPDS2.1* and *qVacAUDPC2.1* were the same, we considered them to be the same locus and named this locus *qVacBrc2.1*.

In regard to 100-seed weight, which was measured in three populations, ICIM identified as many as 11 QTLs for this trait (Table 3 and Figure 3). The QTLs were distributed on LGs 1 to 7. Depending on the population and environment, these QTLs explained 4.80% to 26.84% of the total trait variation and showed additive effects of 0.07 to 0.14 and dominant effects of −0.05 to 0.04.

### 3.5. Comparison of QTLs for Bruchid Resistance

**Figure 5:**
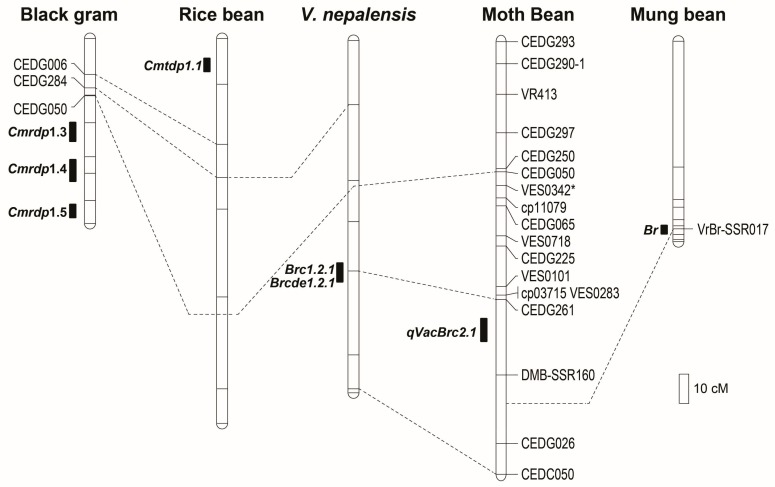
Comparative linkage map of QTLs for seed resistance to *C. chinensis* and *Callosobruchus maculatus* on linkage group 2 of moth bean (*Vigna aconitifolia*; this study), mungbean (*Vigna radiata*), azuki bean (*Vigna nepalensis*), black gram (*Vigna mungo*), and rice bean (*Vigna umbellata*). Dotted lines between maps connect common markers or indicate their possible positions.

Quantitative trait loci *qVacBrc2.1* for *C. chinensis* resistance, detected in moth bean on LG2, was compared with QTLs for bruchid (*C. chinensis* and/or *C. maculatus*) resistance mapped to LG2 in mungbean (*V. radiata*) [20,24], wild azuki bean (*V. nepalensis*) [8], black gram [25], and rice bean [23] even though only a small number of markers were common among linkage maps. BLASTN analysis revealed that genome conservation between moth bean and azuki bean/mungbean, especially azuki bean, was generally very high (Table S1). According to the QTL comparison, QTL *qVacBrc2.1* in moth bean was similar to two QTLs in *V. nepalensis*, namely, *Brc1.2.1* controlling the percentage of damaged seeds and *Brcde1.2.1* contributing to the developmental period (days to emergence) of *C. chinensis*; however, *qVacBrc2.1* was different from *qBr*, a QTL for the percentage of damaged seeds due to *C. chinensis* and *C*. *maculatus* in mungbean (Figure 5). The genomic location of *qVacBrc2.1* differed from that of the QTL for *C. maculatus* resistance in rice bean (Figure 5). Although we were not able to clearly compare *qVacBrc2.1* with the QTL for bruchid resistance in black gram because marker information was insufficient for the latter, *qVacBrc2.1* may be identical to one of the three QTLs for *C. maculatus* resistance in black gram (Figure 5).

## 4. Discussion

Postharvest seed loss due to bruchid infestation is a major problem in moth bean and other crops of the leguminous genus *Vigna*. Genetic study of bruchid resistance in *Vigna* crops, especially at the genome level, has been hindered because these species are minor or underutilized crops grown mainly in developing countries, with a corresponding lack of genomic resources. Recently, however, the whole genomes of mungbean and azuki bean were sequenced and released [21,22]. These resources are useful for genomics studies, both for these two species as well as their congeners, as genomes within *Vigna* are highly conserved [26]. Using QTL mapping and comparative genomic analysis, we were able to compare QTLs for bruchid resistance in different *Vigna* species.

The segregation analysis in this study suggested that seed resistance to *C. chinensis* in wild moth bean accession TN67 is controlled by a single dominant locus. This finding is similar to that of previous genetic studies of seed resistance to bruchids in mungbean, cowpea, and black gram, where bruchid resistance has been found to be a monogenic or oligogenic trait. In cultivated and wild mungbean, for example, resistance to *C. chinensis* and *C. maculatus* is controlled by a single dominant locus (*Br*) [16,27], while resistance to *C. maculatus* in wild black gram is controlled by two dominant duplicated loci (*Cmr1* and *Cmr2*) [28]. In cultivated cowpea, resistance to *C. maculatus* is controlled by one or two recessive loci with modifiers [29]. In our study, QTL analysis uncovered two QTLs for *C. chinensis* resistance in moth bean accession TN67: a major QTL consistently identified in different generations and environments and a minor QTL detected in a specific environment (Table 3 and Figure 4). The results of our QTL analysis confirm the monogenic segregation for the resistance trait observed in the F_2_ and F_2:3_ populations. Consequently, breeding *C. chinensis*-resistant moth bean cultivars should not be difficult. SSR markers CEDG261 and DMB-SSR160 may be used for marker-assisted selection to accelerate the development of such cultivars.

Some physical seed characteristics, such as seed size, have been reported to contribute to bruchid resistance. For example, QTL mapping for *C. chinensis* resistance in a wild azuki bean (*V. nepalensis*) clearly demonstrated a negative relationship between seed size and resistance [8]. In addition, QTL mapping demonstrated the colocalization of the *Br* locus for bruchid resistance with a QTL for seed size in a wild mungbean [30]. In our study, the resistant parent (TN67) and the susceptible parent (IPCMO056) had contrasting seed sizes (1.23 vs. 2.65 g per 100 seeds, respectively). No significant correlation was observed between seed size and the percentage of damaged seeds or AUDPC (developmental period of *C. chinensis*) in the progenies derived from the two parents even though a minor QTL for bruchid resistance (*qVacPDS5.1*) and a minor QTL for seed size (*qVacSDW5.2*) were co-located (Figure 3). This lack of correlation indicates that seed size has no effect on *C. chinensis* resistance in moth bean TN67. The longer developmental period and lower percentage of damaged seeds observed in TN67 also indicate that the resistance is due to antibiosis in seeds rather than seed size or other seed characteristics. This result confirms that no association exists between seed size and resistance in TN67. Our findings in moth bean are the same as a previous determination in rice bean that QTLs for seed size do not associate with those for bruchid resistance [31].

Genome mapping studies of *Vigna* species, including mungbean, azuki bean, rice bean, black gram, and cowpea, have revealed that their genomes are highly similar. Genes/QTLs controlling the same traits in these species have been mapped to similar genomic regions; these mapped traits include seed size, days to flowering, seed dormancy, and pod length [26,32,33,34,35]. Genes/QTLs for bruchid resistance are similarly conserved among *Vigna* species, especially on LG2, where the QTL for resistance in mungbean [20,24], *V. nepalensis* [8], black gram [25], and rice bean [23] has been found to reside. The results of our QTL mapping of *C. chinensis* resistance in moth bean TN67 (Figure 3 and Figure 5) further highlight the strong conservation of this trait on the homologous chromosome. In *Vigna* species, the number and effects of QTLs/genes for bruchid resistance on LG2 are different: one locus (the *Br* locus) conferring very high or complete resistance to both *C. chinensis* and *C. maculatus* is present in mungbean [20,24], three loci with partial resistance to only *C. maculatus* are found in black gram [25], one locus giving partial resistance to only *C. maculatus* is present in rice bean [23], and one locus responsible for partial resistance to only *C. chinensis* is found in *V. nepalensis* [8]. Our QTL mapping and genome comparison revealed one principal QTL, *qVacBrc2.1*, and one modifying QTL, *qVacBrc5.1*, that confer *C. chinensis* resistance in moth bean. *qVacBrc2.1* is likely identical to a QTL for *C. chinensis* resistance in *Vigna nepalensis* and possibly the same as one of three QTLs for *C. maculatus* resistance in wild black gram (Figure 5). Regardless of the identity of these QTLs, our results demonstrate the diverse genetic basis of resistance against *Callosobruchus bruchids* in *Vigna*. Our findings should be useful for sustainable breeding for resistance and also demonstrate the high potential of comparative genomic analysis for identifying genes controlling useful traits in *Vigna*, especially minor or underutilized species.

## Figures and Tables

**Table 1:** Variation and heritability of the percentage of damaged seeds and the area under the disease progress curve (AUDPC) (progress of infestation severity) due to *Callosobruchus chinensis* (azuki bean weevil) in IPCMO056, TN67 and IPCMO056 × TN67 F_2_ and F_2:3_ populations.

Population	Percentage of Damaged Seeds			AUDPC		
	Min–Max	Mean	Heritability (%)	Min–Max	Mean	Heritability (%)
IPCMO056	95.4–100	98.1	-	2826.0–3158.0	2992.0	-
TN67	6.6–21.6	11.9	-	197.0–500.0	348.5	-
F_2_ population	0.0–100	41.6	83.60	0.0–3103.1	1163.2	85.99
F_2:3_ population (2016)	3.5–99.6	57.0	90.42	Not determined		
F_2:3_ population (2017) (Set I)	0.5–100	53.1	96.03	16.4–3385.0	1536.7	98.65
F_2:3_ population (2017) (Set II)	1.0–100	52.2	99.97	Not determined		

**Table 2:** Chi-square test under a single gene model for the percentage of damaged seeds (% damaged seeds) and the AUDPC due to *C. chinensis* in moth bean F_2_ and F_2:3_ populations derived from an IPCMO056 × TN67 cross.

Population	Trait	No. of Plants/Lines Tested	Resistant: Susceptible	Chi-Square (*p* Value)
F_2_	% damaged seeds	187	143:44	0.2157 (0.6423)
	AUDPC	187	142:45	0.0873 (0.7676)
F_2:3_-A ^1^	% damaged seeds	172	125:47	0.4961 (0.4812)
F_2:3_-B^2^ (set I)	% damaged seeds	166	121:45	0.3936 (0.5304)
F_2:3_-B^2^ (set II)	% damaged seeds	166	122:44	0.2880 (0.6541)
	AUDPC	166	116:50	2.3213 (0.1276)

^1^ The F_2:3_ population was grown from February to May 2016. ^2^ The F_2:3_ population was grown from December 2016 to March 2017.

**Table 3:** Quantitative trait loci (QTLs) detected by inclusive composite interval mapping (ICIM) for the percentage of damaged seeds (% damaged seeds) and the AUDPC due to *C. chinensis* and 100-seed weight in moth bean F_2_ and F_2:3_ populations from the cross IPCMO056 × TN67.

Population	Trait	LG ^a^	QTL name	Position ^b^	Franking Markers	LOD	PVE ^c^ (%)	Add ^d^	Dom ^e^
F_2_	% damaged seeds	2	*qVacPDS2.1*	91	CEDG261—DMB-SSR160	69.55	62.98	42.64	−11.16
	AUDPC	2	*qVacAUDPC2.1*	91	CEDG261—DMB-SSR160	82.20	63.00	1273.19	−483.39
	100-seed weight	3	*qVacSDW3.1*	22	VES084—CEDG155	3.72	6.48	0.10	0.01
		4	*qVacSDW4.2*	65	CEDG091—CEDG165	6.38	11.11	0.14	−0.02
		5	*qVacSDW5.1*	0	CEDG020—VES0091	5.28	9.01	0.11	−0.03
		6	*qVacSDW6.2*	71	CEDG146—cp09781	7.25	15.56	0.15	−0.02
F_2:3_-A	% damaged seeds	2	*qVacPDS2.1*	90	CEDG261—DMB-SSR160	40.22	50.41	32.91	2.98
		5	*qVacPDS5.2*	17	CEDG264—VES0664	14.54	12.19	0.51	22.76
	100-seed weight	2	*qVacSDW2.1*	36	CDEG297—CEDG250	4.66	7.93	0.09	−0.01
		3	*qVacSDW3.2*	45	VES0053—CEDG084	7.49	11.82	0.09	0.04
		4	*qVacSDW4.1*	32	*Bms*—VES0675	14.01	26.84	0.14	−0.03
		5	*qVacSDW5.2*	18	VES0664—CEDG027	4.86	7.66	0.07	0.03
		6	*qVacSDW6.1*	20	CEDG169—CEDG034	3.65	5.80	0.67	0.01
F_2:3_-B (Set I)	% damaged seeds	2	*qVacPDS2.1*	91	CEDG261—DMB-SSR160	46.79	61.32	41.42	0.73
	AUDPC	2	*qVacAUDPC2.1*	91	CEDG261—DMB-SSR160	44.90	58.73	1279.74	−90.70
	100-seed weight	1	*qVacSDW1.1*	2	CEDG149—CEDC007	4.51	6.75	0.08	0.03
		3	*qVacSDW3.2*	55	VR169—VES0070	6.98	14.01	0.11	0.01
		4	*qVacSDW4.1*	28	*Bms*—VES0675	8.43	16.03	0.11	−0.05
		4	*qVacSDW4.2*	63	CEDG091—CEDG165	4.22	6.24	0.07	−0.01
		5	*qVacSDW5.1*	0	CEDG020—CEDG091	3.61	4.80	0.06	−0.01
		7	*qVacSDW7.1*	19	CEDG174—CEDG215	5.23	8.46	0.83	0.01
F_2:3_-B (Set II)	% damaged seeds	2	*qVacPDS2.1*	91	CEDG261—DMB-SSR160	47.97	64.23	40.98	0.10

^a^ LG: Linkage group; ^b^ position on the linkage group (centimorgans); ^c^ phenotypic variance explained by the QTL; ^d^ additive effect; ^e^ dominant effect.

## Supplementary Materials

The following are available online at http://www.mdpi.com/2073-4425/9/11/555/s1, Table S1: Location of DNA markers mapped to linkage group 2 (LG2) of moth bean F2 population (IPCMO056 × TN67) on the reference genome of mungbean ([21]; http://plantgenomics.snu.ac.kr) and azuki bean ([22]; http://viggs.dna.affrc.go.jp).
